# Fucoidan Extract Enhances the Anti-Cancer Activity of Chemotherapeutic Agents in MDA-MB-231 and MCF-7 Breast Cancer Cells 

**DOI:** 10.3390/md11010081

**Published:** 2013-01-09

**Authors:** Zhongyuan Zhang, Kiichiro Teruya, Toshihiro Yoshida, Hiroshi Eto, Sanetaka Shirahata

**Affiliations:** 1 Department of Bioscience and Biotechnology, Graduate School of Bioresource and Bioenvironmental Sciences, Kyushu University, 6-10-1 Hakozaki, Higashi-ku, Fukuoka 812-8581, Japan; E-Mails: zzyszy2012@yahoo.co.jp (Z.Z.); sirahata@grt.kyushu-u.ac.jp (S.S.); 2 Faculty of Agriculture, Kyushu University, 6-10-1 Hakozaki, Higashi-ku, Fukuoka 812-8581, Japan; 3 Yoshida Clinic, 6-18-27 Higashi Mikuni, Yodogawa-ku, Osaka 532-0002, Japan; E-Mail: info@yoshidaiin.net; 4 Daiichi Sangyo Co., Ltd., 6-7-2 Nishitenman, Kita-ku, Osaka 530-0037, Japan; E-Mail: ask_daiichisangyo@daiichi-sangyo.com

**Keywords:** fucoidan extract, chemotherapeutic agents, anti-cancer activity, apoptosis, breast cancer

## Abstract

Fucoidan, a fucose-rich polysaccharide isolated from brown alga, is currently under investigation as a new anti-cancer compound. In the present study, fucoidan extract (FE) from *Cladosiphon navae-caledoniae* Kylin was prepared by enzymatic digestion. We investigated whether a combination of FE with cisplatin, tamoxifen or paclitaxel had the potential to improve the therapeutic efficacy of cancer treatment. These co-treatments significantly induced cell growth inhibition, apoptosis, as well as cell cycle modifications in MDA-MB-231 and MCF-7 cells. FE enhanced apoptosis in cancer cells that responded to treatment with three chemotherapeutic drugs with downregulation of the anti-apoptotic proteins Bcl-xL and Mcl-1. The combination treatments led to an obvious decrease in the phosphorylation of ERK and Akt in MDA-MB-231 cells, but increased the phosphorylation of ERK in MCF-7 cells. In addition, we observed that combination treatments enhanced intracellular ROS levels and reduced glutathione (GSH) levels in breast cancer cells, suggesting that induction of oxidative stress was an important event in the cell death induced by the combination treatments.

## 1. Introduction

Natural dietary compounds have been widely and safely consumed over centuries, and preclinical studies suggest that most of them have potential applications in pharmacology and cancer therapy. Fucoidan is a naturally occurring polysaccharide compound in brown alga, such as *Fucus vesiculosus*, *Cladosiphon okamuranus* and *Laminaria saccharina* [1,2,3]. Studies *in vitro* have indicated that fucoidan provides protection against various cancers, including human lymphoma, promyelocytic leukemia, colon carcinoma, breast carcinoma, hepatoma and melanoma [4,5,6,7,8,9]. It was found that fucoidan inhibits angiogenesis of melanoma, and it has anti-metastatic activity against Lewis lung adenocarcinoma and 13762 MAT rat mammary adenocarcinoma in mouse xenograft models [10,11,12]. Clinical studies have shown that fucoidan causes tumor regression and subjective improvement of overall survival in cancer patients [13]. These findings confirm the efficacy of fucoidan against human cancers. Fucoidan exerts pleiotropic effects on cancer cells involving the induction of apoptosis through caspase-cascade activation, regulation of c-Jun *N*-terminal kinase (JNK), extracellular signal-regulated kinase (ERK) and p38 signaling, Bcl-2 protein expression and Akt signaling, inhibition of angiogenesis by suppressing vascular endothelial growth factor expression and inhibition of cell transformation induced by epidermal growth factor receptor [2,4,5,6,7,14,15]. In our previous studies, we showed that fucoidan extract (FE) induced apoptosis in MCF-7 cancer cells via ROS-dependent JNK activation and a mitochondrion-mediated pathway [8]. Since fucoidan has anti-tumor properties both *in vitro* and *in vivo*, a combination of fucoidan with chemotherapeutic drugs might be an intriguing option in the therapy of cancer patients. Ikeguchi *et al.* [16] have performed a clinical trial in patients with unresectable advanced or recurrent colorectal cancer. The patients who received 150 mL/day of fucoidan were able to endure prolonged chemotherapy without fatigue. The survival of patients with fucoidan treatment was longer than that of patients without fucoidan treatment, although the difference was not significant [16]. Therefore, the application of combination approaches involving chemotherapeutic agents could improve drug absorption and enhance the clinical response.

Low molecular weight FE was used in this study, which was obtained by enzymatic digestion of a high molecular weight FE purified from *Cladosiphon navae-caledoniae* Kylin. The digested low molecular weight FE is more water-soluble than undigested high molecular weight FE, which affects absorption *in vivo* and, thus, bioavailability [17,18,19,20]. Cisplatin (CDDP) is a widely used chemotherapeutic agent for various types of cancers. It has been confirmed that CDDP exerts its cytotoxicity by interference with transcription or DNA replication mechanisms, leading to cell cycle checkpoint activation and sustained G2 arrest [21]. CDDP has been reported to cause apoptosis mediated by the activation of distinct signal pathways, including death receptor signaling, mitogen-activated protein kinases (MAPKs) signaling, protein kinase Akt signaling, p53 signaling and the activation of mitochondrial pathways [22]. Tamoxifen (TAM) is a selective estrogen receptor (ER) antagonist that is extensively used in the treatment of both advanced-stage and early-stage estrogen receptor-positive breast cancers [23]. Clinical response to TAM has been shown to be associated with both decreased proliferation and increased apoptosis. Mechanisms associated with apoptosis have been described, including production of oxidative stress and activation/expression of modulation proteins, such as ERK and JNK, transforming growth factor-β, protein kinase C, as well as Bcl-2 protein family members [24]. Paclitaxel (TAXOL), a natural chemotherapeutic drug isolated from the bark of the pacific yew, is currently used in the treatment of breast cancer and ovarian cancer. TAXOL-treated cancer cells undergo cell cycle arrest and apoptosis [25]. The activities of TAXOL have been described and include effects on cell signaling and gene expression, activation of MAPKs, Raf-1, protein tyrosine kinases and regulation of Bcl-2-related proteins, such as Bcl-2, Bcl-xL and Bad [26,27]. 

The data presented here show that low molecular weight FE in combination with CDDP, TAM or TAXOL significantly enhanced cell death of MDA-MB-231 and MCF-7 breast cancer cells by regulating the expression of Bcl-2 family proteins, modulating ERK and Akt signaling and regulating the production of oxidative stress.

## 2. Results and Discussion

### 2.1. Enhanced Cytotoxicity by Combination of FE and Chemotherapeutic Agents

**Figure 1:**
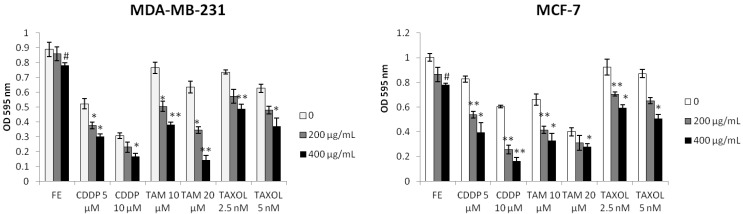
Synergistic inhibition of cell growth. Cell proliferation was analyzed using the MTT assay. Cells were treated with fucoidan extract (FE), Cisplatin (CDDP), Tamoxifen (TAM) or Paclitaxel (TAXOL) alone or in combination for 48 h (*n* = 5). Results represent the mean ± SD from three independent experiments. A significant difference from control is indicated by *p* < 0.05 (^#^) or *p* < 0.01 (^##^); a significant difference from single treatments is indicated by *p* < 0.05 (*) or *p* < 0.01 (**).

MDA-MB-231 and MCF-7 breast cancer cells were exposed to FE or FE plus one of the three commonly used chemotherapeutic agents, namely, CDDP, TAM or TAXOL. In the absence of chemotherapeutic agents, FE exhibited a dose-dependent cytotoxicity to the cells (Figure 1). MCF-7 cells were much more sensitive than MDA-MB-231 cells. Among the three agents, MCF-7 cells were relatively resistant to CDDP treatment and relatively sensitive to TAM treatment compared to MDA-MB-231 cells. In the presence of 400 μg/mL FE, more than 80% cell growth inhibition was induced by 10 μM CDDP in both MDA-MB-231 and MCF-7 cells. FE also enhanced TAM-induced (20 μM) cell growth inhibition from 29% to 84% in MDA-MB-231 cells. When FE was combined with TAXOL, inhibition of MCF-7 cell growth was similar to that of MDA-MB-231 cells. The synergistic effect of FE on chemotherapeutic agent-induced cell proliferation reduction was dose-dependent.

### 2.2. Synergistic Induction of Apoptosis and Cell Cycle Arrest by FE and Chemotherapeutic Agents

**Figure 2:**
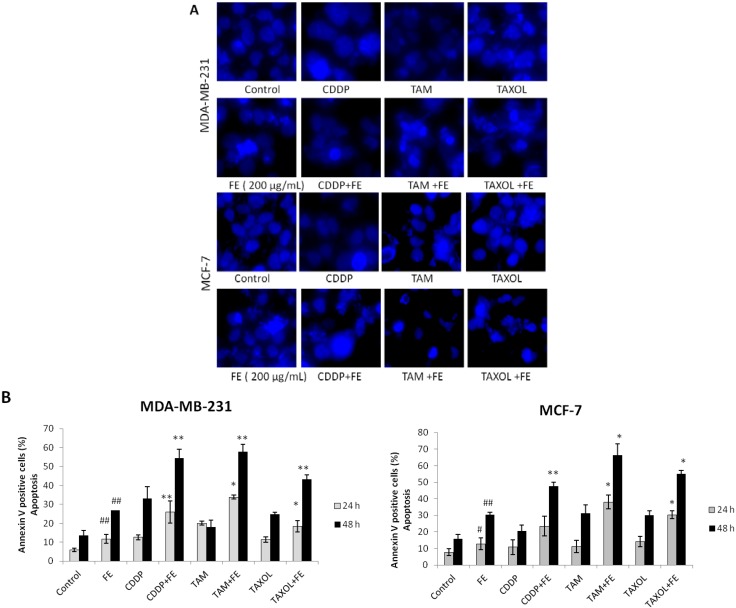
Synergistic induction of apoptosis by co-treatment. (**A**) Hoechst 33342 staining of cells treated with 200 μg/mL FE alone or 200 μg/mL FE in combination with 5 μM CDDP, 10 μM TAM or 2.5 nM TAXOL for 48 h. Each experiment shown is representative of 20 randomly observed fields; (**B**) Analysis of apoptotic cells by annexin/PI double-staining using the IN Cell Analyzer 1000. MDA-MB-231 and MCF-7 cells were treated for different times with 200 μg/mL FE alone or 200 μg/mL FE in combination with 5 μM CDDP, 10 μM TAM or 2.5 nM TAXOL. All results were obtained from three independent experiments. A significant difference from control is indicated by *p* < 0.05 (^#^) or *p* < 0.01 (^##^); a significant difference from single treatments is indicated by *p* < 0.05 (*) or *p* < 0.01 (**).

To determine if the combination treatment-induced cytotoxicity occurred through necrosis and/or apoptosis, cells were assayed with Hoechst 33342 staining and annexin V-FITC staining. As shown in Figure 2A, in untreated cells, nuclear chromatin was homogeneously dispersed throughout the whole nucleus. When combining FE with chemotherapeutic agents, cells exhibited a shrunken nucleus, peripheral clumping and nuclear fragments with bright chromatin. Enhanced apoptosis of tumor cells by the combination treatments was evident by an increased number of annexin V-positive cells in a time-dependent manner (Figure 2B). We found that 200 μg/mL FE enhanced 5 μM CDDP-induced apoptosis from 32.9% to 52.4% in MDA-MB-231 cells and from 20.4% to 47.6% in MCF-7 cells. Combining FE with TAM (10 μM) enhanced cell death from 17.9% to 57.9% in MDA-MB-231 cells and from 31% to 66% in MCF-7 cells after 48 h of treatment. Apoptotic cell death increased about two-fold over 48 h in the presence of both FE and TAXOL (2.5 nM) relative to TAXOL alone in both MDA-MB-231 cells and MCF-7 cells. 

We next performed flow cytometry analysis to investigate changes in cell cycle distribution upon co-treatment. As shown in Table 1, CDDP alone or in combination with FE strongly induced the accumulation of MDA-MB-231 and MCF-7 cells in the G2/M phase. The combination of both FE and TAM slightly increased the number of MCF-7 cells in the G1 phase and had little effect on the cell cycle distribution in MDA-MB-231 cells. TAXOL or combination treatment in both cell lines caused an increase in the number of cells in the G2/M phase. The percentage of cells in the sub-G1 phase was significantly increased by the three co-treatments in the two cell lines, suggesting that the combination of two agents induced cell death more effectively than any single agent did.

**Table 1:** Analysis of cell cycle distribution. Cells were treated with FE, CDDP, TAM or TAXOL alone or in combination with FE for 48 h. Cells were then measured by flow cytometry as described in the Methods section. The cell distribution was analyzed by flow cytometry analysis software.

Treatment	MDA-MB-231					MCF-7			
	G1	S	G2/M	Sub-G1		G1	S	G2/M	Sub-G1
Control	65.9 ± 2.8	21.9 ± 2.0	12.2 ± 1.8	1.9 ± 0.2		54.6 ± 5.2	31.7 ± 2.5	13.7 ± 0.5	5.0 ± 0.2
FE (200 μg/mL)	68.7 ± 1.0	18.8 ± 1.6	12.5 ± 0.9	10.7 ± 0.8		57.3 ± 4.8	27.5 ± 1.5	15.2 ± 1.2	13.6 ± 1.0
CDDP (5 μM)	13.7 ± 0.3	29.4 ± 3.5	56.9 ± 3.2	17.5 ± 4.3		37.1 ± 1.4	14.1 ± 1.0	48.8 ± 1.5	8.9 ± 1.5
CDDP + FE	19.9 ± 0.7	21.4 ± 0.6	58.7 ± 0.4	37.8 ± 6.2		26.2 ± 2.5	29.0 ± 2.6	44.8 ± 2.0	32.4 ± 3.6
TAM (10 μM)	66.6 ± 3.6	23.3 ± 2.1	10.1 ± 1.6	4.9 ± 0.6		63.7 ± 2.2	18.9 ± 1.5	17.4 ± 0.9	12.6 ± 0.9
TAM + FE	60.8 ± 2.0	22.7 ± 1.5	16.5 ± 1.5	18.3 ± 2.1		67.8 ± 2.4	17.6 ± 1.6	14.6 ± 1.1	46.5 ± 4.3
TAXOL (2.5 nM)	54.5 ± 2.0	20.5 ± 1.5	25 ± 0.8	6.4 ± 0.4		54.2 ± 2.2	16.9 ± 1.3	29.1 ± 0.8	8.4 ± 0.6
TAXOL + FE	53.7 ± 1.9	22.1 ± 2.1	24.2 ± 1.6	25.8 ± 2.4		49.8 ± 2.6	21.8 ± 1.0	28.4 ± 0.7	20.6 ± 0.3

### 2.3. Synergistic Changes in the Expression of Bcl-2 Proteins

**Figure 3:**
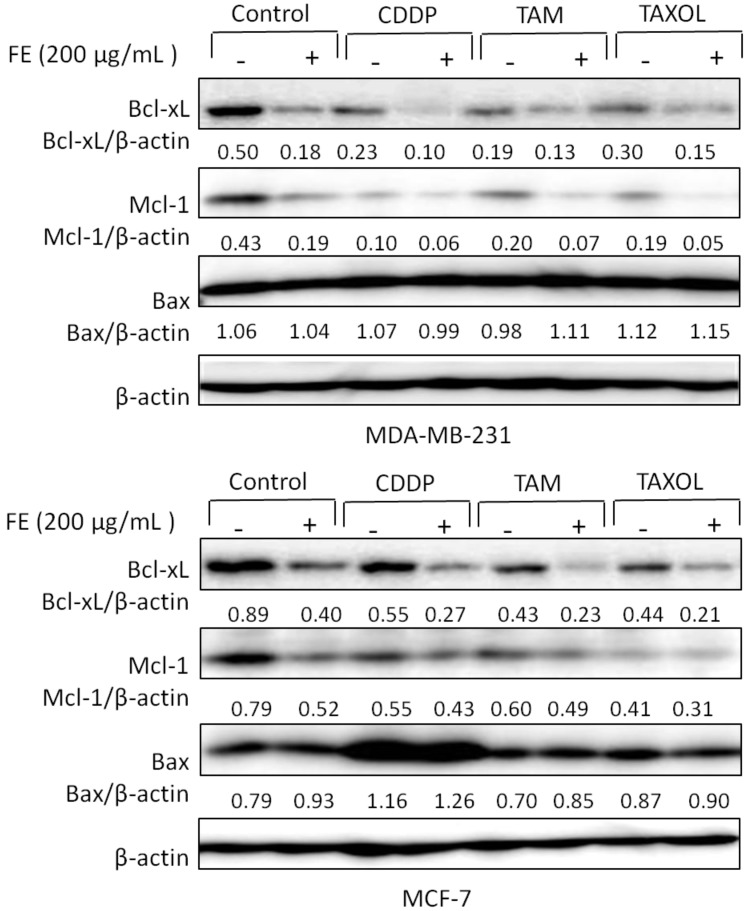
Regulation of expression of Bcl-2 proteins by combination treatments. MDA-MB-231 and MCF-7 cells were treated with 200 μg/mL FE or 200 μg/mL FE in combination with 5 μM CDDP, 10 μM TAM or 2.5 nM TAXOL for 48 h. The expression of Bcl-xl, Mcl-1 and Bax was determined by Western blotting using specific antibodies. Experiments were conducted three times; representative blots are shown.

To investigate the cell death mechanism induced by the combination treatments, Western blot analysis of the breast cancer cells was performed. Our previous experiments revealed that FE affected the regulation of expression of Bcl-2 family proteins [8]. Therefore, we first tested whether co-treatment with chemotherapeutic agents could further regulate the effect of FE on the expression of Bcl-2 proteins. The data in Figure 3 show that 200 μg/mL FE alone efficiently reduced the expression of the anti-apoptotic proteins Bcl-xL and Mcl-1 in both MDA-MB-231 and MCF-7 cells. In combination, CDDP, TAM and TAXOL further reduced Bcl-xL and Mcl-1 expression. Significant up-regulation of the expression of the pro-apoptotic protein Bax was observed only in MCF-7 cells and was observed only with FE, CDDP or combination treatment.

### 2.4. Modulations in ERK and Akt Phosphorylation

**Figure 4:**
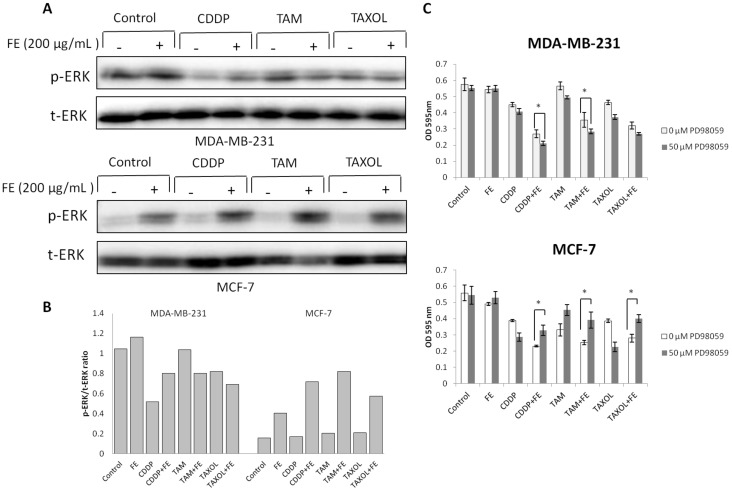
Modulation of ERK phosphorylation by combination treatments. (**A**) MDA-MB-231 and MCF-7 cells were treated with FE or FE in combination with CDDP, TAM or TAXOL for 48 h. ERK1/2 and its phosphorylated forms were determined by Western blotting using specific antibodies; (**B**) The relative amount of protein expression was quantified using Quantity One software (Bio-Rad) and normalized by the band intensity of total ERK; (**C**) MDA-MB-231 and MCF-7 cells were pretreated with 50 μM PD98059 for 1 h and then exposed to FE or FE in combination with CDDP, TAM or TAXOL for 48 h. After treatment, MTT was used to analyze cell survival. All results were obtained from three independent experiments. Differences with *p* < 0.05 (*) or *p* < 0.01 (**) were considered statistically significant.

We next determined the effects of co-treatment on the levels of survival receptor ERK. The data in Figure 4A,B show that combination treatments partially decreased the phosphorylation of ERK in MDA-MB-231 cells compared with untreated controls or FE treatment alone. Unexpectedly, an obvious increase in ERK phosphorylation was observed by the addition of FE in MCF-7 cells; this change was further increased by combination treatments. Among the three drugs, FE in combination with TAM resulted in a four-fold increase in ERK phosphorylation, compared to the untreated control. To evaluate the role of ERK phosphorylation in the induction of cell death, MCF-7 and MDA-MB-231 cells were treated with 50 μM PD98059 prior to their exposure to the agents (Figure 4C). We found that PD98059 not only increased the extent of cell death induced by CDDP, TAM or TAXOL alone, but also increased the cell death caused by combination treatment in MDA-MB-231 cells. Interestingly, in the presence of PD98059, the combination of FE and CDDP, TAM or TAXOL resulted in partially decreased levels of cell death in MCF cells. A combination of FE and TAM induced an approximately 50% increase in cell viability. 

**Figure 5:**
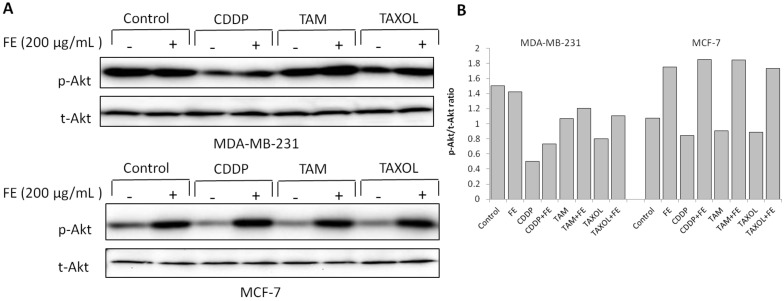
Modulation of Akt phosphorylation by combination treatments. (**A**) MDA-MB-231 and MCF-7 cells were treated with FE or FE in combination with CDDP, TAM or TAXOL for 48 h. Akt and its phosphorylated forms were determined by western blotting using specific antibodies; (**B**) The relative amount of protein expression was quantified using Quantity One software and normalized by the band intensity of total Akt.

Activation of the Akt signaling pathway has been reported to mediate drug resistance and promote survival of cancer cells [28]. Therefore, we examined the status of Akt after drug treatment. As shown in Figure 5, FE alone produced an increase in the level of Akt phosphorylation in MCF-7 cells, with little change in Akt phosphorylation in MDA-MB-231 cells. Combination of FE plus CDDP, TAM or TAXOL exhibited inhibition of Akt phosphorylation compared with FE treatment alone in MDA-MB-231 cells. Phosphorylation of Akt was suppressed to approximately 50% by treatment of the cells with CDDP plus FE relative to FE treatment alone (Figure 5B).

### 2.5. Synergistic Increases in ROS Levels and Reduction in GSH Levels

**Figure 6:**
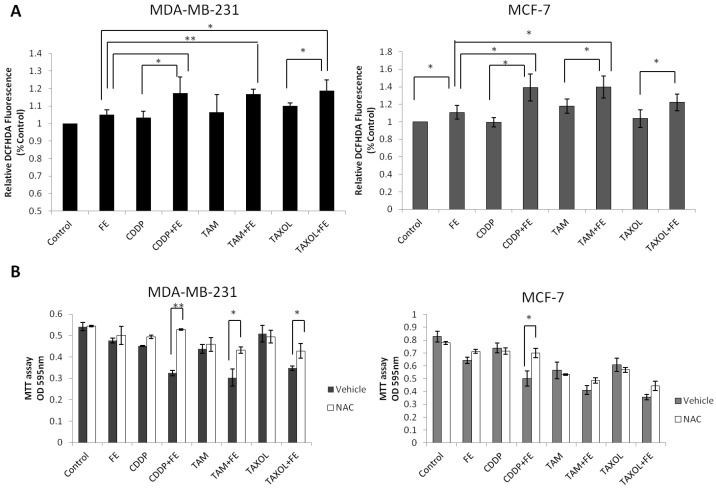
Synergistic increases in ROS levels. (**A**) MDA-MB-231 and MCF-7 cells were treated with FE or FE in combination with CDDP, TAM or TAXOL for 48 h. Then, the cells were labeled with 5 μM DCFH-DA and the fluorescence was measured using the IN Cell Analyzer 1000, as described in the Methods section; (**B**) MDA-MB-231 and MCF-7 cells were pretreated with 5 mM NAC for 1 h and then exposed to FE or FE in combination with CDDP, TAM or TAXOL for 48 h. After treatment, MTT was used to analyze cell survival. All results were obtained from three independent experiments. Differences with *p* < 0.05 (*) or *p* < 0.01 (**) were considered statistically significant.

Our previous study showed that FE induced ROS generation in MCF-7 cells. To examine the effect of combination treatments on ROS generation, DCFH-DA was used as a molecular probe. As shown in Figure 6A, treatment of MDA-MB-231 cells with FE alone had a slight impact on ROS generation, similar to that in MCF-7 cells. The combination treatment with FE and the chemotherapeutic agents led to enhanced generation of ROS in the two cell lines. To determine whether ROS contributed to cell death, we tested the effect of NAC on drug-treated cancer cells (Figure 6B). As expected, pretreatment with NAC partially attenuated the cell cytotoxicity induced by FE in combination with chemotherapeutic agents in MDA-MB-231 cells. However, in MCF-7 cells, significant inhibition of cell cytotoxicity was observed only with a combination of FE and CDDP after treatment with NAC.

**Figure 7:**
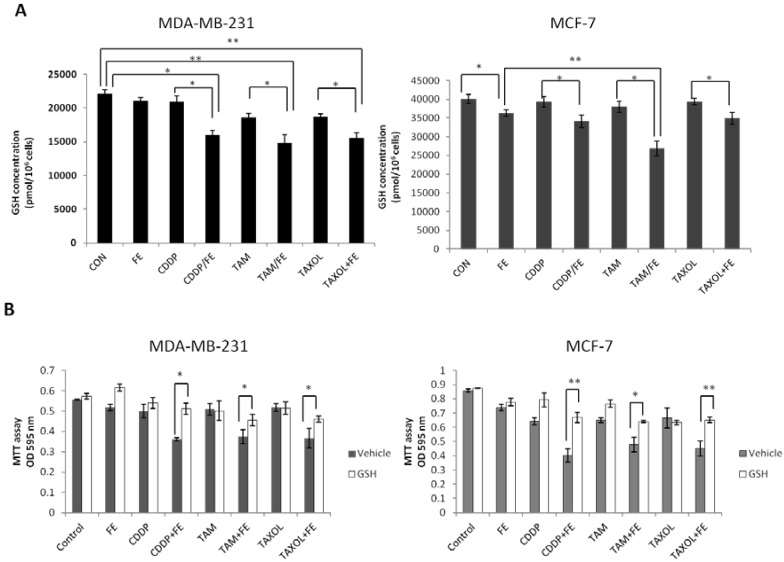
Synergistic reduction of GSH levels. (**A**) MDA-MB-231 and MCF-7 cells were treated with FE or FE in combination with CDDP, TAM or TAXOL for 48 h. Total GSH levels were quantified using the GSH Assay Kit (Trevigen Inc., Gaithersburg, MD, USA); (**B**) MDA-MB-231 and MCF-7 cells were incubated with FE or FE in combination with CDDP, TAM or TAXOL for 48 h in the presence or absence of 5 mM GSH; cell survival was analyzed using the MTT assay. All results were obtained from three independent experiments. Differences with *p* < 0.05 (*) or *p* < 0.01 (**) were considered statistically significant.

Oxidative stress can be facilitated by reduced intracellular GSH levels that promote apoptosis. Therefore, we measured total GSH levels in the breast cancer cells exposed to FE or in combination with chemotherapeutic agents (Figure 7A). A slight, but statistically significant, reduction in GSH levels of 10.5% was observed for FE-treated MCF-7 cells. FE in combination with the chemotherapeutic agents enhanced the reduction of GSH levels more than chemotherapeutic agents alone in the two cell lines. Treatment with the combination of FE and TAM further reduced GSH levels by up to 31.5% in MDA-MB-231 cells and by 33% in MCF-7 cells. Furthermore, the intracellular GSH content was replenished by the addition of externally supplied GSH. As shown in Figure 7B, in the presence of 5 mM GSH, a large increase in cell viability was observed in the cells treated with a combination of FE and chemotherapeutic agents.

### 2.6. Global Discussion

A growing body of evidence indicates that fucoidans function as prospective anti-cancer agents *in vitro*, as well as *in vivo*. However, the *in vivo* concentration achieved after oral administration of fucoidan is much lower than the concentration that demonstrated efficacy *in vitro* [29]. Although the reported efficacy of fucoidan may be achieved by the application of higher oral or intravenous doses, it has the potential to be an anti-cancer drug based on its synergistic activity with other chemotherapeutic drugs.

In the present study, we investigated the combination of FE and three chemotherapeutic drugs, which exhibit highly synergistic inhibition effects on cancer cell growth, thus allowing the effective concentration of FE to be reduced relative to our previous study. We noted that FE significantly enhanced cell proliferation reduction induced by CDDP, TAM or TAXOL alone in MDA-MB-231 and MCF-7 breast cancer cells. Morphological changes of the nucleus and the number of time-dependent annexin V-positive cells increased, indicating that the combination treatments caused apoptotic cell death. FE itself did not arrest cell cycle progression in MCF-7 cells in our previous study [8]. However, the combination treatment induced modifications in the cell cycle distribution in both MDA-MB-231 and MCF-7 cells, suggesting additive or synergistic effects occurred with the co-treatment.

In this study, the combination treatments relative to FE or chemotherapeutic drugs alone partially reduced Bcl-xL and Mcl-1 expression in MDA-MB-231 and MCF-7 cells. It has been reported that concomitant downregulation of Mcl-1 and Bcl-xL is sufficient to induce massive cell death in human mesothelioma cells [30], and reduction of Bcl-xL expression caused reduction of tumor growth *in vivo*, as well as increased survival in mouse models [31]. Furthermore, pro-apoptotic Bak is sequestered by Mcl-1 and Bcl-xL; therefore, inactivation of these proteins by BH3-only proteins is necessary and sufficient for Bak-mediated cell death [32]. These findings indicate that Mcl-1 and Bcl-xL downregulation may serve as a convergence point for inducing massive apoptotic cell death and avoiding therapeutic failure of FE in combination with chemotherapeutic drug treatments. In addition, our previous studies showed that FE treatment induced mitochondrion-dependent apoptosis in MCF-7 cells, as demonstrated by the regulation of Bcl-2 proteins and the release of cytochrome c and AIF. The results presented in this study regarding regulation of Mcl-1, Bcl-xL and Bax expression in cells treated by the combination therapy is likely to cause the induction of Bcl-2 protein-mediated, mitochondrion-dependent cell death. Further experiments will be required to determine if the mitochondria is the major target for the FE-enhanced increased efficacy of chemotherapy.

ERK activation induced by fucoidan has been shown to contribute to the signal transduction pathways leading to cell apoptosis [4,6,15]. We have previously demonstrated that ERK phosphorylation occurred rapidly and persisted for a period after FE exposure in MCF-7 cells [8]. The present study characterized the effects of a combination of FE and chemotherapeutic agents on cell death with respect to ERK activation. Surprisingly, our data showed that a combination of FE and chemotherapeutic agents decreased ERK phosphorylation induced by FE in MDA-MB-231 cells; however, it was increased in MCF-7 cells. Furthermore, pretreatment with PD98059 promoted cell death in MDA-MB-231 cells treated by combination therapy, but blocked cell death in similarly treated MCF-7 cells. These results suggest that a combination of FE and chemotherapeutic agents modified the ERK activation induced by FE and demonstrated different effects on transduction pathways in MDA-MB-231 cells and MCF-7 cells. It has been reported that fucoidan exerts activation of the ERK1/2 signaling pathway associated with cell apoptosis in HL-60 cells and HCT-15 cells [6,15]. On the other hand, the effect of fucoidan on ERK inhibition was observed in HS-Sultan cells [4]. These findings indicate that it is likely that chemotherapeutic agents exhibit various synergistic effects on cell death due to cell type-specific factors. MEK/ERK activity has been shown to act on mitochondrion-dependent apoptosis by modulating expression of Bcl-2 family proteins, such as Bax and Bcl-xL, and they regulate cell cycle arrest associated with cyclin D1 transcription and cyclin B/cdc2 nuclear translocation [33,34,35]. Our studies concerning the involvement of cell cycle arrest and apoptosis in combination treatments indicated that ERK phosphorylation might mediate cell cycle arrest and apoptosis in breast cancer cells. In addition, mechanisms of estrogen-mediated cellular actions have been shown to be very complex [36]. TAM has been shown to activate ERK rapidly in ER-positive MCF-7 and T47D cells, but not significantly in ER-negative MDA-MB-231 cells, which demonstrates a correlation between activated ERK and ER expression in the induction of rapid death of breast cancer cells by TAM [37]. Doxorubicin has been shown to decrease the phosphorylation of ERK in ER-negative MDA-MB-231 cells; however, phosphorylation was increased in ER-positive MCF-7 cells [38]. It is possible that FE and in combination with anti-cancer drugs can activate different receptor and signaling pathways in ER-negative MDA-MB-231 cells compared to ER-positive MCF-7 cells.

There is abundant evidence that activation of Akt signaling mediates cancer cell growth and may serve as a potential target for drug development. Fucoidan has been reported to inhibit Akt phosphorylation in lymphoma HS-Sultan cells [4]. However, significant inhibition of Akt phosphorylation in FE-treated breast cancer cells was not observed in the present study. We noted that combination treatment involving FE and anti-cancer drugs reduced Akt phosphorylation in MDA-MB-231 cells, but had no effect in MCF-7 cells. Cross-talk between the Ras-Raf-MEK-ERK and PI3K-Akt pathways has been demonstrated in several cellular systems [34,39,40]. It is possible that disruption of Akt signaling has a greater impact on cell death under conditions of MEK/ERK inactivation, because the combination treatment resulted in decreased ERK phosphorylation in MDA-MB-231 cells. It has been reported that estrogen promotes association of ERK with the IGF-1 receptor and the p85 subunit of PI3-kinase in the plasma membrane, which leads to AKT activation [41,42]. In addition, estrogen-mediated Akt activation has been reported to be blocked by the estrogen antagonist ICI 182780 in ER-positive MCF-7 cells [43]. The discrepancy between the two cell lines might be attributed to differences in ER status. Inactivation of Akt seems to be important, at least in the apoptotic response of ER-negative breast cancer cells against combination treatments. Activation of Akt was observed in ER-positive MCF-7 cells, suggesting that FE might act as an ER agonist to stimulate Akt. This hypothesis suggests a negative impact of FE as a single drug on ER-positive breast cancer patients, but a potential positive interaction when combined with chemotherapeutic drugs for ER-negative breast cancer patients. However, further *in vivo* studies are required to address this possibility.

Cellular redox status or oxidative stress has been shown to be involved in cell proliferation, growth arrest or apoptotic cell death based on the extent of redox imbalance [44]. In our study, combination treatments somewhat enhanced ROS generation induced by FE in both MDA-MB-231 and MCF-7 cells. Pretreatment with NAC partially increased cell viability for all combination treatments in MDA-MB-231 cells, suggesting that ROS generation is related to cell death. Meanwhile, in MCF-7 cells, significant inhibition of cell cytotoxicity failed to be observed with the combination of FE and TAM or the combination of FE and TAXOL after treatment with NAC. It is possible that ROS generation is not necessary for cell death by these two combination treatments in MCF-7 cells. High intracellular GSH levels have been associated with apoptotic resistance, and GSH depletion itself has been found to trigger cell death cascades [45]. We observed that GSH was further reduced with combination treatment relative to FE treatment alone. The addition of external GSH to the medium protected against cell death. These findings suggest that oxidative stress is involved in the progression of cell death caused by a combination of FE with the three chemotherapeutic agents. In addition, early GSH depletion has been reported to cause dissipation of the ΔΨm with simultaneous ROS generation prior to induction of apoptosis [46]. We previously found that ROS is involved in mitochondria dysfunction in FE-treated MCF-7 cells [8]. It is likely that generation of intracellular ROS and depletion of GSH are related to the induction of mitochondria-dependent cell death in breast cancer cells. Thus, these findings support the hypothesis that oxidative stress plays a role as a common mediator of cell death by treatment with a combination of FE and chemotherapeutic agents.

## 3. Experimental Section

### 3.1. Cell Culture

Estrogen receptor (ER)-positive MCF-7 and ER-negative MDA-MB-231 cells were obtained from the American Type Culture Collection (Manassas, VA, USA). Cells were maintained in DME medium supplemented with 10% fetal bovine serum in an incubator in a humidified atmosphere of 5% CO_2_ at 37 °C.

### 3.2. Materials

The abalone glycosidase-digested FE was generously donated for this study by the Daiichi Sangyo Corporation (Osaka, Japan). FE was prepared as previously described [2]. Briefly, high molecular weight fucoidan extract was purified to 85% from seaweed of *Cladosiphon navae-caledoniae* Kylin and digested with glycosidases to obtain FE. FE consists of a digested small molecular weight fraction (72%, MW < 500 Da) and non-digested fractions (less than 28%, peak MW: 800 kDa). FE consisted of mostly fucose (73%), xylose (12%) and mannose (7%). The ratio of sulfation was 14.5%. 

Cisplatin, tamoxifen citrate, paclitaxel, 3-(4,5-dimethyl-2-thiazolyl)-2,5-diphenyl-2*H*-tetrazolium bromide (MTT) and propidium iodide (PI) were purchased from Wako Pure Chemical Industries, Ltd. (Osaka, Japan). *Cellstain*^®^-Hoechst 33342 was obtained from DOJINDO Laboratories (Kumamoto, Japan). The annexin V-FITC apoptosis kit was obtained from Medical and Biological Laboratories Co., Ltd. (Nagoya, Japan). PD98059 and 2′,7′-dichlorofluorescin diacetate (DCFH-DA) were purchased from Merck KGaA (Darmstadt, Germany). The ROS inhibitor NAC was purchased from Sigma-Aldrich Co. (St. Louis, MO, USA). The GSH Assay Kit was purchased from Trevigen Inc. (Gaithersburg, MD, USA). All antibodies used in the study were purchased from Cell Signaling Technology, Inc. (Danvers, MA, USA).

### 3.3. MTT Assay

Cells were seeded in 96-well plates at densities of 2000 cells/well for 24 h. The cells were then incubated with FE or chemotherapeutic drugs plus FE. After 48 h of incubation, 10 μL of MTT solution (5 mg/mL) was added to each well and incubated for an additional 4 h at 37 °C. The medium was aspirated and replaced with 200 μL/well of DMSO to dissolve the formazan salt. The color intensity of the formazan solution was measured at 595 nm using a microplate reader (Tecan Group Ltd., Männedorf, Switzerland).

### 3.4. Hoechst Staining

Cells were seeded onto 96-well plates (5 × 10^3^ cells/well) and cultured for 24 h. After 48 h of treatment with drugs, cells were washed with PBS and stained with 2 μg/mL Hoechst 33342 for 15 min. Images were then captured with the IN Cell Analyzer 1000 (GE Healthcare UK Ltd., Buckinghamshire, UK). Each image shown is representative of 20 randomly observed fields.

### 3.5. Annexin V Binding Assay

FITC-labeled annexin V was applied and analyzed by the IN Cell Analyzer 1000. After drug treatment, cells were stained with the annexin V-FITC solution (annexin V-fluorescein in binding buffer containing PI and Hoechst 33342). The cells were further incubated for 15 min in the dark, and then, images of the cells were acquired using the IN Cell Analyzer 1000. Cells with apoptotic morphology of the nuclei (condensation/fragmentation) or annexin V-positive cells were analyzed using the Developer Toolbox software (GE Healthcare UK Ltd.). For each analysis, 3000 cells were recorded. 

### 3.6. Flow Cytometry Analysis

Cell cycle distribution was performed by flow cytometry. After treatment, the cells were collected by trypsin, washed in cold PBS and fixed with 70% ethanol on ice for 30 min. The fixed cells were washed with PBS and incubated in 10 μg/mL RNase A for 30 min at room temperature. PI (10 μL of 1 mg/mL) was added to the cell suspension and cells were incubated for 10 min in the dark. The DNA content was analyzed by flow cytometry (Beckman Coulter, Inc., Brea, CA, USA). The proportion of cells in G1, S and G2/M phases was determined using FlowJo software (Tree Star, Inc., Ashland, OR, USA). For each sample, 1 × 10^4^ cells were recorded.

### 3.7. Western Blot Analysis

Cell protein lysates were prepared and Western blot analyses were performed as described previously [8]. The proteins were separated by SDS-polyacrylamide gel electrophoresis. The proteins were then transferred onto polyvinylidene difluoride membranes (GE Healthcare UK Ltd.) and blotted with each antibody. Protein bands were visualized using enhanced chemiluminescence, as described by the supplier (GE Healthcare UK Ltd.).

### 3.8. Measurement of Intracellular ROS

To detect generation of intracellular ROS, cells were incubated with drugs for 5 h and labeled with 5 μM DCFH-DA at 37 °C for 30 min. Then, the cellular fluorescence intensity was measured with the IN Cell Analyzer 1000 after washing the cells twice with PBS. For each sample, 3000 events were recorded. Image analysis was carried out using the IN Cell Investigator software using either the Developer Toolbox or the Multi-Target Analysis (MTA) Module (GE Healthcare UK Ltd.).

### 3.9. GSH Assay

The GSH concentration was measured by the GSH Assay Kit, according to the manufacturer’s instructions. Briefly, the drug-treated cells were collected in cold 5% metaphosphoric acid. The samples were sonicated and then incubated on ice for 5 min. The suspension was then centrifuged at 12,000× *g* for 5 min at 4 °C. Total GSH content was measured with a microplate reader at 405 nm.

### 3.10. Statistical Analysis

Each experiment was performed at least in triplicate and repeated three times. The results are presented as the mean ± standard deviation (SD) values. The difference between the two groups was analyzed using the two-tailed Student’s *t*-test; differences among three or more groups were analyzed by one-way analysis of variance multiple comparisons.

## 4. Conclusions

In conclusion, the present study demonstrates that FE, an enzymatically digested polysaccharide, cooperates with the chemotherapeutic agents CDDP, TAM and TAXOL to induce cell growth inhibition through the induction of apoptosis and cell cycle arrest in the human MDA-MB-231 and MCF-7 breast cancer cell lines. These studies highlight the potential regarding the achievable efficacy of FE in combination with chemotherapeutic agents in cancer treatment. Further *in vivo* and clinical studies are needed to evaluate the safety and utility of these combination treatments in cancer patients.
